# A 68‐year‐old man with gait instability and T2 signal abnormality in the cerebellar peduncles

**DOI:** 10.1111/bpa.13172

**Published:** 2023-06-07

**Authors:** Vanessa L. Smith, Shih‐Hsiu J. Wang

**Affiliations:** ^1^ Department of Pathology Duke University Medical Center Durham North Carolina USA; ^2^ Department of Pathology and Department of Neurology Duke University Medical Center Durham North Carolina USA

**Keywords:** FMR1, FXTAS, neurodegenerative

BOX 1 **Virtual glass slide**

Access at https://isn‐slidearchive.org/?col=ISN&fol=Archive&file=BPA‐22‐11‐279.svs

## 1 CASE DESCRIPTION

**FIGURE 1:**
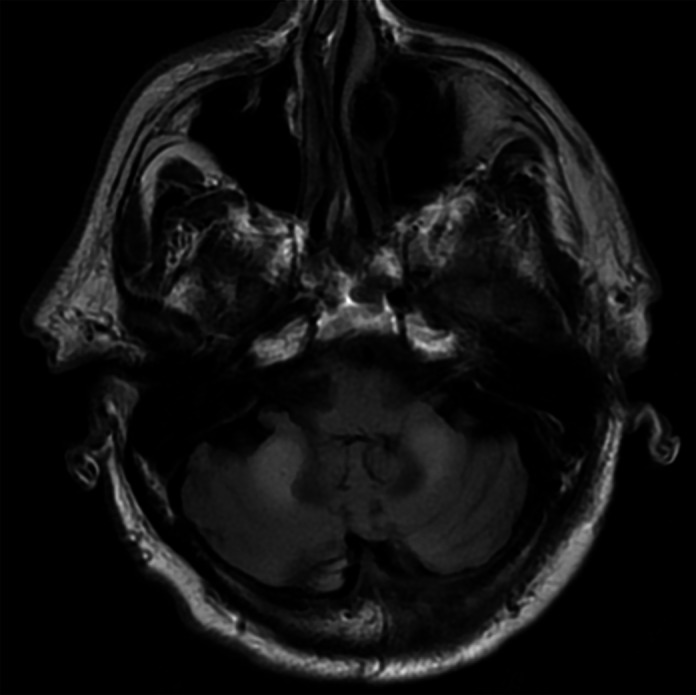
Magnetic Resonance Imaging on FLAIR sequence demonstrates symmetric FLAIR signal abnormality in the cerebellar peduncles bilaterally.

The patient first came to clinic at 68 years old with a complaint of gait instability and longstanding weakness in his bilateral lower extremities. On exam, he had mild bilateral lower extremity spasticity with quadriceps hyperreflexia. MRI revealed nonenhancing foci of hyperintense T2 signal abnormality in the periventricular and subcortical white matter particularly involving the left precentral gyrus. High T2 and FLAIR signal abnormality was also present in the pons and cerebellar peduncles bilaterally (Figure 1). At 70, he began to complain of tremor in his hands and memory decline. Neurological exam showed cogwheeling, a large amplitude tremor, and wide‐based gait. He returned at 77 for follow up of slowly worsening tremor, gait and balance issues, and memory difficulty. He now had hallucinations on the junction between sleep and wakefulness, raising the possibility of Parkinsonian neurodegeneration, though a trial of carbidopa‐levodopa did not ameliorate his symptoms. He continued to have slowly worsening symptoms until death at age 82. An autopsy was performed.

## 2 FINDINGS

**FIGURE 2:**
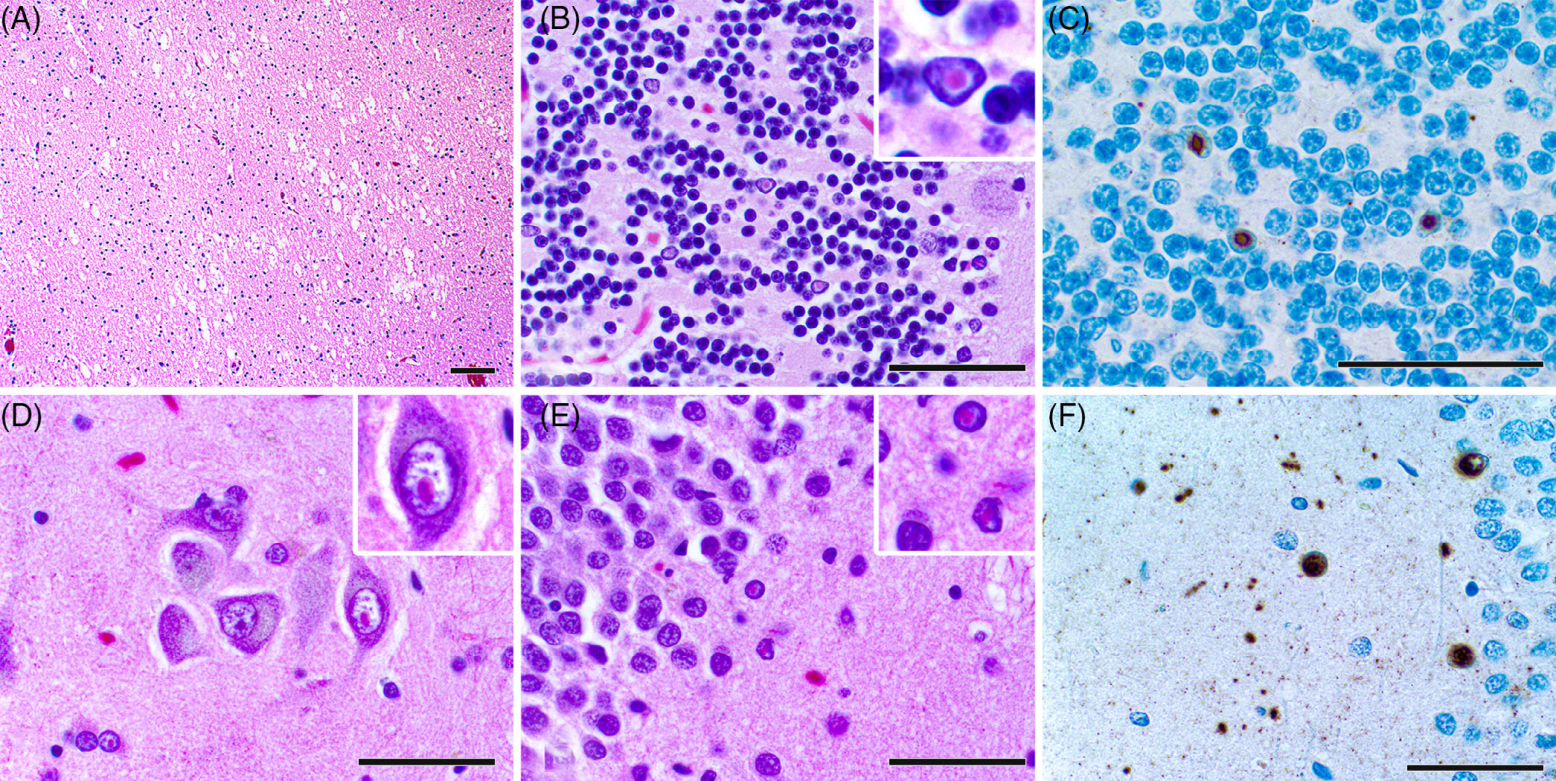
H&E stained slides of the cerebellum (A–C) and hippocampus (D–F) demonstrate patchy spongiosis (A) at low power and intensely eosinophilic intranuclear inclusion bodies (B, D, E) at high power. An immunostain for p62 is positive in some nuclei, consistent with the presence of intranuclear inclusion bodies (C, F). Scale bar = 100 μm (A) and 50 μm (B–F).

Gross examination of the brain showed moderate dilation of the lateral ventricles and cavum septum pellucidum. Also, there was a 0.6 × 0.3 cm well‐circumscribed gelatinous and soft area of the corpus callosum. Microscopic examination shows spongiosis in this latter area and patchy areas of spongiosis in the subcortical and cerebellar white matter (Figure 2A, Box 1). Luxol fast blue (LFB) and neurofilament (NFP) immunostain demonstrates loss of myelinated axons in these areas. There is patchy neuronal loss in the hippocampus and Purkinje cell dropout with Bergman gliosis in the cerebellum. On high power, brightly eosinophilic round to ovoid intranuclear inclusions are present in the cerebellar granular layer (Figure 2B) and hippocampus (Figure 2D,E), often with a peri‐inclusion halo. Immunostain for p62 highlights numerous intranuclear inclusions in the cerebellum (Figure 2C), hippocampus (Figure 2D), entorhinal cortex, and adjacent temporal neocortex.

## 3 DIAGNOSIS

Fragile X‐associated Tremor/Ataxia Syndrome.

Genetic testing for *FMR1* CGG repeat expansion revealed 87 (+/− 3) CGG repeats.

## 4 DISCUSSION

The *FMR1* gene contains a CGG repeat in the 5′ untranslated region (5′UTR) of the gene. When more than 200 repeats are present, the promoter and trinucleotide repeat region become hypermethylated, resulting in gene silencing. This results in fragile X syndrome, the leading heritable cause of intellectual disability.

Because the *FMR1* gene silencing is resultant of trinucleotide repeat expansion, it can exhibit genetic anticipation manifesting as premutation disorders. While gene silencing only occurs with expansions greater than 200 repeats, expansions of less than 200 CGG repeats result in a paradoxical increase in gene activity. The prevailing theory is that this increase in mRNA levels leads to partial sequestration of at least one RNA‐binding protein, resulting in functional insufficiency of the protein(s) and cellular toxicity [1].

Two premutation disorders have been described for *FMR1*: Fragile X‐associated primary ovarian insufficiency (FXPOI) and Fragile X‐associated tremor/ataxia syndrome (FXTAS). FXPOI occurs in female carriers of *FMR1* premutation and results in irregular menstrual cycles, infertility, early menopause, and elevated follicle stimulating hormone. FXTAS is thought to occur primarily in males secondary to the protective effects of the second X chromosome in females and results in progressive ataxia, tremor, memory loss, peripheral neuropathy, and mental and behavioral changes. It usually develops late in life and worsens over time due to age‐dependent penetrance [1, 2].

On imaging, FXTAS can present as nonspecific, subcortical, patchy regions of increased T2/FLAIR signal intensity in the cerebrum. In a high percentage, increased T2/FLAIR signal intensity is present in the middle cerebellar peduncles and can also be seen in the deep cerebellar white matter and brainstem.

On gross examination, the cerebrum and cerebellum demonstrate broad white matter disease. Mild to moderate cortical atrophy and ventriculomegaly are also present, along with brainstem atrophy that is most notable in the pons [2].

Microscopically, the finding of neuronal intranuclear inclusion bodies is characteristic. These inclusion bodies appear as discrete, hyaline‐appearing, eosinophilic, round to ovoid bodies that are 2–5 μm in diameter and are single within a nucleus, except in Purkinje cells. They stain for ubiquitin and p62 but are negative for periodic acid‐Schiff (PAS), silver, tau, and neurofilament. Additionally, Purkinje cell loss beyond that expected with otherwise normal aging and axonal swellings/torpedoes are common. Typically, Bergmann gliosis accompanies Purkinje cell loss. In the white matter of both the cerebrum and cerebellum, abnormal areas demonstrate spongiosis, axonal degeneration, and myelin loss. In the most severe cerebral white matter changes, scattered fibrillary astrocytes are greatly enlarged by irregular expansion of cytoplasm that contains lysosomal debris. The middle cerebellar peduncles may also show myelin pallor on LFB–PAS stain [1, 2].

While a full understanding of both the mechanisms of penetrance and of pathogenesis is still under investigation, the inclusion of FXTAS in the differential of patients with Parkinsonian neurodegenerative disease is important. Identification allows for appropriate treatment of the patient, as well as for informed health decisions of the patient's family who may have inherited the premutation.

## AUTHOR CONTRIBUTIONS

Vanessa L. Smith wrote the original draft. Shih‐Hsiu J. Wang made the neuropathological diagnosis and reviewed and edited the draft.

## CONFLICT OF INTEREST STATEMENT

The authors declare no conflict of interest.

## ETHICS STATEMENT

All data related to this case are deidentified.

## Data Availability

Data sharing is not applicable to this article as no new data were created or analyzed in this study.
